# Surgical site infection after 769 Tibial Plateau Leveling Osteotomies

**DOI:** 10.3389/fvets.2023.1133813

**Published:** 2023-04-13

**Authors:** Benjamin Husi, Gudrun Overesch, Franck Forterre, Ulrich Rytz

**Affiliations:** ^1^Clinic for Small Animal Surgery, Vetsuisse Faculty, University of Zurich, Zurich, Switzerland; ^2^Department of Infectious Diseases and Pathobiology, Institute of Veterinary Bacteriology, Vetsuisse Faculty, University of Bern, Bern, Switzerland; ^3^Division of Small Animal Surgery, Department of Clinical Veterinary Medicine, Vetsuisse Faculty, University of Bern, Bern, Switzerland

**Keywords:** multidrug-resistant bacteria, MDR, SSI, surgical site infection, Tibial Plateau Leveling Osteotomy, TPLO

## Abstract

**Objective:**

To report surgical site infections (SSI) after Tibial Plateau Leveling Osteotomy (TPLO), treatment course, associated risk factors, bacterial isolates and antimicrobial resistance.

**Study design:**

Retrospective clinical cohort study.

**Study population:**

Six hundred and twenty seven dogs and 769 TPLO procedures.

**Methods:**

Data from electronic medical records of dogs undergoing TPLO between 2005 and 2015 at a single institution have been retrospectively reviewed. A generalized mixed logistic regression was used to determine possible risk factors. The Chi-Square test of independence was used to examine the relationship between the isolation of multidrug-resistant (MDR) bacteria and the development of major infections undergoing additional surgical treatment. To assess the correlation between number of SSI and number MDR isolate per year, Pearson's correlation coefficient was calculated.

**Results:**

The overall complication rate was 19.3% (*n* = 149). SSI was most frequent with 8.5% (*n* = 65). Major SSI occurred in 6.8% (*n* = 52) TPLO (80.0% SSI). *Staphylococcus* (*S*.) *pseudintermedius* (*n* = 37) and *S*. *aureus* (*n* = 10) were most frequently isolated. Multidrug-resistant bacteria were identified in 2.7% (*n* = 21) TPLO (32.3% SSI) but were not associated with major SSI (*p* = 0.426). There was a strong positive correlation between number of MDR isolates per year and number of SSI per year [*r*_(9)_ = 0.79, *p* = 0.004]. Factors associated with SSI were previous TPLO in the contralateral stifle (*p* = 0.02, OR = 2.01, 95% CI = 1.11–3.64) and German Shepherd dogs (*p* = 0.035, OR = 4.41, 95% CI = 1.11–17.54). The use of non-locking implants was found to be protective (*p* = 0.02, OR = 0.179, 95% CI = 0.18–0.77).

**Clinical significance:**

Infection with multidrug-resistant bacteria is an emerging problem in veterinary practice and treatment is challenging. The incidence of major SSI was found to be high but was not associated with the isolation of MDR bacteria.

## 1. Introduction

Tibial Plateau Leveling Osteotomy (TPLO) is one of the most commonly performed surgical procedures for the treatment of cranial cruciate ligament (CCL) disease in dogs (1, 2). Postoperative complication rates in the literature range from 9.7% to 27.8% (3–7). The reported incidence of surgical site infections (SSI) ranges from 2.9% to 17.3% (4, 5, 8–14) and is considered higher than expected in comparison to other sterile elective orthopedic procedures (15). The large variation in the literature can partially be explained by different definitions of key terms used for postoperative complication and infection. Due to the extensive usage of TPLO in veterinary surgery, SSI has a great economic impact on pet owners and influences patients' recovery from a surgery that is considered a standard procedure (16). Previous publications have suggested a variety of factors associated with SSI after TPLO including surgeon's experience, increased body weight, duration of anesthesia, breed predisposition for German Shepherd Dogs, and use of non-locking implants for dogs >50 kg (4, 9, 12, 14, 17).

The emergence of multidrug-resistant (MDR) bacteria is widely acknowledged and poses a major therapeutic challenge in both human and veterinary medicine (18, 19). Literature reports a prevalence of 20**–**40% multidrug-resistant *Staphylococcus (S*.) *aureus* and *S. pseudintermedius* isolates among dogs with SSI after TPLO (8, 10, 11, 16, 20, 21). For human patients suffering from implant-associated MRSA-infection, the Centers for Disease Control and Prevention (CDC) guidelines recommend long-term systemic antimicrobial therapy, surgical debridement, and, whenever possible, removal of the surgical implants (22). In veterinary medicine, the selection of antimicrobials is limited since many of the compounds remaining effective are considered of critical importance for treatment of infections with MDR bacteria in human medicine and should therefore be avoided (23). Consequently, effective systemic antimicrobial treatment is sometimes not possible. Furthermore, bacteria commonly involved in SSI in dogs, in particular *Staphylococcus* spp., are known to form biofilms that hinder hosts' immune response and effectively prevent appropriate concentrations of antimicrobials at the site of infection (24). For this reason, removal of orthopedic implants is often considered necessary but, in the case of TPLO, is only feasible when the degree of bone healing at the osteotomy site guarantees appropriate stability.

The aim of this retrospective study was firstly to analyze risk factors for SSI after TPLO and report bacterial isolates and their antimicrobial resistance pattern. Secondly, we sought to investigate the influence of occurrence of multidrug-resistant bacteria on treatment of SSI.

## 2. Materials and methods

### 2.1. Data collection

A retrospective cohort study was conducted. Medical records of dogs that underwent stifle arthroscopy and TPLO in a veterinary teaching hospital between January 2005 and December 2015 were reviewed. The minimum duration between data collection and surgical procedure was set to 6 months. Procedures were excluded, if medical records were incomplete or other orthopedic procedures were simultaneously performed on the same stifle apart from arthroscopic treatment of meniscal injury. Medical records were considered incomplete, if information on signalment, documentation of surgical procedure and anesthesia, or postoperative care was missing. Available follow up information was extracted from medical records in the form of documentation of in-house rechecks or communication logs. If no postoperative complications were recorded, the recovery was considered uneventful. Dogs undergoing bilateral staged TPLO were considered independent cases and the statistical model was designed to account for the dependence on multiple observations of a single animal by modeling a random intercept for each animal. Data collection included breed, age, sex, body weight, history of dermatitis, use of locking or non-locking implant, duration of anesthesia, pre-, intra-, and postoperative antimicrobial administration, and if previous contralateral TPLO had been performed (during or before the study period). In cases of postoperative complications, the following data was collected: Type and timepoint of complication, type of therapy and outcome. Results of bacteriological culture and antimicrobial susceptibility testing were reviewed.

### 2.2. Surgery

Arthroscopy was used for inspection of the stifle joint before every TPLO. Remnants of ruptured CCL were debrided, and meniscal damage treated with a combination of meniscal knife, motorized shaver, and arthroscopic punch forceps. TPLO as described by Slocum and Slocum (25) was performed using a jig. Osteotomy was stabilized using adequately sized TPLO standard or broad plates by Slocum Enterprises (Eugen, Oregon, USA), New Generation Devices (Glen Rock, New Jersey, USA), Synthes (Synthes Vet, West Chester, Pennsylvania, USA) or Fixin (TraumaVet, Rivoli, Piedmont, Italy) depending on surgeon's preference and availability. Cefazolin (20 mg/kg iv.) was administered within 60 min before surgery and repeated every 90 min until the end of the procedure. Postoperative antimicrobials were not routinely prescribed.

### 2.3. Definition of surgical site infection

Criteria for diagnosis of SSI were based on those published by the United States Centers for Disease Control and Prevention (CDC) (26), with minor modifications. One or more of the following criteria had to be met: (1) Purulent drainage from the incision site. (2) Spontaneous wound dehiscence or wound reopened by surgeon AND fever, localized pain, OR tenderness, unless the incision was cultured negative. (3) Abscess, fistula, or other evidence of infection on clinical examination, diagnostic imaging, surgery, cytology, or histopathology. (4) Signs of inflammation AND positive bacterial culture of an aseptically collected sample from the surgical site. Based on the categorization of Cook et al. (27), with minor adjustments, SSI were further defined as catastrophic, major and minor. A SSI was considered a catastrophic complication if it resulted in permanent unacceptable function or was directly related to death or euthanasia. A major SSI was defined as requiring additional surgical intervention. In contrast to Cook and colleagues, infections treated only medically were defined as minor. This modification is in consensus with previous studies on SSI after TPLO (4, 5, 9).

### 2.4. Bacterial cultures and antimicrobial susceptibility testing

Bacterial cultures and antimicrobial susceptibility testing (AST) were performed at the Institute of Veterinary Bacteriology (IVB) at the Vetsuisse Faculty, University of Bern. Bacterial cultivation of swab/aspirate and implant samples followed standard diagnostic procedures. Identification of colonies were performed by Vitek™ 2 Compact technology (BioMérieux, Marcy l'Etoile, France) until 2010. Since 2011, isolates were identified by Matrix-Assisted-Laser-Desorption/Ionization-Time-of-Flight-Mass-Spectroscopy (MALDI-TOF MS) (Microflex LT, Bruker Daltonics GmbH, Bremen, Germany).

From 2005 until May 2010, AST was performed using ATP-Strips (BioMérieux) and interpretation of test results followed manufacturers' instructions. Since August 2010, AST was performed with the Vitek™ 2 Compact technology (BioMérieux). Isolates were classified as susceptible or resistant, according to clinical breakpoints published by the European Committee on Antimicrobial Susceptibility Testing (EUCAST) guidelines. If no EUCAST breakpoints were available, veterinary clinical breakpoints of the Clinical and Laboratory Standards Institute (CLSI) were used. For *Pasteurella* spp. The above-mentioned methods were not applicable, therefore detection of beta-lactamase enzymatic activity using BBL™ DrySlide™ Nitrocefin (BD Becton Dickinson) was used. Bacteria were evaluated for multidrug resistance (MDR) according to criteria published by the European Center for Disease Control (ECDC) and Centers of Disease Control (CDC) (non-susceptible to ≥1 agent in ≥3 antimicrobial categories as described) (28).

### 2.5. Statistical analysis

Descriptive data were reported as mean ± standard deviation or median (range) as appropriate. Normality of data was assessed using Shapiro-Wilk Test. Statistical analyses were performed by use of statistical software (R Core Team [2015], R Foundation for Statistical Computing, Vienna, Austria). A generalized mixed logistic regression was fitted to the data with SSI as the response variable and sex, age, body weight, ASA (American Society of Anaesthesiologists Classification) grading, pre- and postoperative antimicrobial therapy, previous contralateral TPLO, duration of anesthesia, implant (locking or non-locking) and breed included as fixed predictors. Breed was defined as a categorical variable with a separate category for the most represented breeds (mixed breed, Labrador, Golden Retriever, Bernese Mountain Dog, Boxer, Rottweiler, Great Dane, German Shepherd). The remaining breeds were summarized in one category (other). The variance inflation index was investigated and was found to be between 1 and 2, hence no problematic correlations among independent variables were identified. The identifier of an animal (ID) was included as a random intercept to account for the dependence on multiple observations of a single animal (bilateral staged surgery). Significance was set as *p* < 0.05. Pearson's Chi-Square test was used to test for independence between the presence of MDR bacteria and the presence of major infection. To assess the correlation between number of SSI and number MDR isolate per year, pearson's correlation coefficient was calculated.

## 3. Results

### 3.1. Population

During the study period, 797 TPLO procedures were recorded. Twenty-eight (*n* = 28) procedures were excluded due to incomplete medical records. A total of 627 dogs were included in the study population, 142 (22.5%) of which underwent staged bilateral TPLO, resulting in total of 769 TPLO procedures. In addition to the bilateral staged procedures performed within the study population, four (*n* = 4, 0.5%) cases were found to have undergone TPLO before the study period, leading to a total of 146 (19.0%) cases with a history of previous TPLO surgery on the contralateral stifle. The study population consisted of 402 (52.3%) neutered and 83 (10.8%) intact females, and 187 (24.3%) neutered and 97 (12.6%) entire males. The mean age was 5.9 ± 2.7 years, and median body weight 35.8 kg (range 10–87 kg). The most common breeds were mixed breed (*n* = 170, 23.1%), Labrador Retriever (*n* = 108, 14.0%), Golden Retriever (*n* = 88, 11.5%), Bernese Mountain Dog (*n* = 54, 7.0%), Boxer (*n* = 38, 4.9%), Rottweiler (*n* = 20, 2.6%), Great Dane (*n* = 18, 2.3%) and German Shepherd (*n* = 17, 2.2%). Only seven (0.9%) dogs had ongoing antimicrobial therapy at the time of surgery for reasons unrelated to TPLO surgery. Follow up information was available after 494/769 (64.2%) procedures. Mean time to last follow up was 247 days (min. 10 days, max. 3,029 days). A summary of patient's demographics with and without SSI can be found in Table 1.

**Table 1:** Demographics of patients undergoing 769 TPLO with and without SSI.

		**SSI (*n* = 65)**	**No SSI (*n* = 704)**
Breed			
	Mixed breed	13 (20.0%)	157 (22.3%)
	Labrador retriever	8 (12.3%)	100 (14.2%)
	Golden retriever	4 (6.2%)	84 (11.9%)
	Bernese mountain dog	5 (7.9%)	49 (7.0%)
	Boxer	2 (3.1%)	36 (5.1%)
	Rottweiler	0 (0.0%)	20 (2.8%)
	Great Dane	0 (0.0%)	18 (2.6%)
	German shepherd	7 (10.8%)	10 (1.4%)
	Other breeds	24 (36.9%)	232 (32.0%)
Sex			
	Male	11 (16.9%)	86 (12.2%)
	Male neutered	19 (29.2%)	168 (23.9%)
	Female	8 (12.3%)	75 (10.7%)
	Female spayed	27 (41.5%)	375 (53.3%)
Age (months) (mean ± SD)		67.1 ± 34.5	70.9 ± 31.9
Bodyweight (kg) (mean ± SD)		36.4 ± 10.9	35.7 ± 11.5
Preoperative antibiotic treatment		1 (1.54%)	6 (0.9%)
Postoperative antibiotic treatment		6 (9.2%)	65 (9.2%)
Previous TPLO of contralateral limb		19 (29.2%)	127 (18.0%)
Implant type			
	Non-locking	2 (3.1%)	122 (17.3%)
	Locking	63 (96.9%)	582 (82.7%)
Duration of anesthesia (min.) (mean ± SD)		246.2 ± 46.8	256.5 ± 49.4

TPLO, Tibial Plateau leveling osteotomy; SSI, surgical site infection.

### 3.2. Surgical procedures

In 55.5% (*n* = 427) of procedures, TPLO was completed on the left stifle, and 44.5% (*n* = 342) on the right. All surgeries were performed by, or under the supervision of, an experienced board-certified surgeon. A complete rupture of the CCL was diagnosed during 472 (61.5%) procedures, a partial rupture during 297 (38.6%). Partial meniscectomy was necessary in 298 stifles (38.5%), of which 284 (95.0%) affected the medial, 12 (4.0%) the lateral and three (1.0%) both menisci. Total meniscectomy was performed on the medial meniscus in three cases and medial meniscal release procedure in seven. Mean duration of anesthesia was 255 ± 49 min. In 71 (9.2%) cases, antibiotic treatment was continued postoperatively. The rational of continued post-operative antibiotic treatment of these cases was found to be history of or suspected ongoing local dermatitis in 13/71 (18.3%) cases, continuation of pre-operative antibiotic treatment in 4/71 (5.6%), minor breach of sterility during the surgical procedure in 2/71 (2.8%) and unknown in 52/71 (73.2) cases. Non-locking implants were used in 124/769 (16.1%) cases and locking in 645/769 (83.9%). The distribution of usage of locking and non-locking implants over time is presented in Table 2.

**Table 2:** Usage of non-locking implants, occurrence of SSI and isolation of MDR bacteria of 769 TPLO between 2005 and 2015.

**Time period**	**Year**	**Total number of TPLO (*n*)**	**Non-locking plates used (*n*)**	**Non-locking plates used (%)**	**SSI (*n*)**	**SSI/TPLO (%)**	**MDR (*n*)**	**MDR/TPLO/(%)**
**2005/2006**		**144**	**101**	**70.1**	**5**	**3.5**	**1**	**0.7**
	2005	69	65	94.2	1	1.4	0	0.0
	2006	75	36	48.0	4	5.3	1	1.3
**2007–2015**		**625**	**23**	**3.7**	**60**	**9.6**	**20**	**3.2**
	2007	60	6	10.0	1	1.7	0	0.0
	2008	73	9	12.3	6	8.2	1	1.4
	2009	93	0	0.0	5	5.4	2	2.2
	2010	74	0	0.0	8	10.8	4	5.4
	2011	82	3	3.7	5	6.1	1	1.2
	2012	70	1	1.4	14	20.0	8	11.4
	2013	58	3	5.2	10	17.2	1	1.7
	2014	47	1	2.1	4	8.5	0	0.0
	2015	68	0	0.0	7	10.3	2	2.9
**2005–2015**		**769**	**124**	16.1	**65**	**8.4**	**21**	**2.7**

TPLO, Tibial Plateau leveling osteotomy; SSI, surgical site infection.

The bold values represent the total number of TPLO in each cathegory in the respective time frame (eg. in 2005/2006, 65 + 36 non-locking plates were used, which a total of 101).

### 3.3. Postoperative complications

An overview of encountered postoperative complications can be found in Table 3. Incidence of SSI was 8.5% (*n* = 65). Median duration between surgery and diagnosis of SSI was 14 days (range 4–855). Catastrophic SSI occurred after one TPLO (0.1% overall, 1.5% SSI). Major SSI was diagnosed in 52/65 (80.0%) SSI. Surgical implants were removed in 42/65 (64.6%). Median duration between surgery and implant removal was 132 days (range: 51–2,858 days). Antimicrobial therapy was initiated or continued after 15/42 explantation procedures. Overall, after 57/65 (87.7%) procedures with SSI antimicrobial therapy was prescribed, consisting of cefalexin (20 mg/kg BID po), amoxicillin/clavulanic acid (12.5 mg/kg BID po), clindamycin (5.5 mg/kg BID po), enrofloxacin (10 mg/kg SID po) or a combination of these. Median duration of antimicrobial treatment was 22 days (range: 5–52 days). According to our data, dogs that had previous TPLO on the contralateral stifle (*p* = 0.02, OR = 2.01, 95% CI = 1.11–3.64) and German Shepherd Dogs (*p* = 0.035, OR = 4.41, 95% CI = 1.11–17.54) were more likely to develop SSI. Furthermore, use of non-locking-implants was associated with lower odds of infection (*p* = 0.02, OR = 0.18, 95% CI = 0.18–0.77). Postoperative antibiotic treatment was not associated with development of SSI (*p* = 0.61).

**Table 3:** Overview of postoperative complications of 769 Tibial Plateau Leveling Osteotomies.

**Complication**	**Number (*n*)**	**Percentage (%)**	**Median (range) time to diagnosis (days)**
Surgical site infection	65	8.4%	14 (4–855)
Subsequent meniscal injury	49	6.4%	194 (37–2,827)
Persistent lameness^a^	17	2.2%	51 (71–595)
Seroma	8	1.2%	7 (3–20)
Tuberositas tibia fracture	6	0.8%	15 (7–49)
Screw breakage	2	0.3%	Not applicable (45–65)
Patellar tendinitis	1	0.1%	21
Total	148	19.2%	62 (3–2,827)

a Patients showing persistent lameness >50 days after TPLO without evident cause.

Incidence of SSI involving MDR bacteria was found to be 2.7% (*n* = 21/769) of TPLO or 32.3% (*n* = 21/65) of SSI. One catastrophic complication occurred; A dog was euthanized upon owner's decision seven days post-surgery after being diagnosed with multiple complications including SSI with isolation of MDR bacteria and tuberositas tibia fracture. Major infection occurred after 18/21 (85.7%). Implants were removed in 14/21 (66.7%) cases, seven of which had been treated with adequate antimicrobials according to AST (Table 4). Comparing infection with MDR bacteria to infection with non-MDR bacteria, no association was found between isolation of MDR bacteria and development of major infection (*p* = 0.426). Absolute and relative number of SSI and SSI involving MDR bacteria is shown in Table 2. There was a strong positive correlation between number of MDR isolates per year and number of SSI per year [*r*_(9)_ = 0.79, *p* = 0.004].

**Table 4:** Treatment and proportion of major SSI by isolated bacteria after 55 TPLO with positive bacterial culture results.

**Isolation of MDR bacteria**	**Bacterial species**	**Total SSI (*n*)**	**SSI with antimicrobial therapy^a^/total SSI (*n*/*n*)**	**Major SSI/total SSI (*n*/*n*)**	**Plate removal/total SSI (*n*/*n*)**
**SSI with MDR bacteria**		**21** ^**b**^	**10/21**	**19/21**	**14/20** ^**c**^
	***S. pseudintermedius***	13	5/13	12/13	9/13
	***S. aureus***	5^b^	3/5	4/5	3/4^c^
	**Other bacterial species**	4^b^	2/4	3/4	2/3^c^
**SSI without MDR bacteria**		**35**	**24/35**	**27/35**	**22/35**
	***S. pseudintermedius***	22	14/22	18/22	17/22
	***S. aureus***	5	4/5	4/5	3/5
	**Other bacterial species**	8	6/8	5/8	2/8
**Total**		**55**	**34**	**46**	**36**

SSI, surgical site infection; S, *Staphylococcus*; MDR, multi-drug resistant bacteria; SSI, surgical site infection;

a Antimicrobial therapy received according to antibiotic suceptibility testing,

b One dog diagnosed with SSI (MDR *Staphylococccus aureus* and MDR *Acinetobacter baumanii*)

c was exluded because of euthanasia shortly after initiation of treatment.

The bold values respresent the total number of cases in the respective category.

After 17 (2.2%) TPLO procedures, persistent lameness >50 days after TPLO without any evident cause was diagnosed. Six of seventeen dogs (35.3%) underwent conservative treatment consisting of non-steroidal anti-inflammatory drugs and restricted activity (*n* = 6, 35.3%), antimicrobial therapy with cefalexin 20 mg/kg for 7–10 days (*n* = 2, 11.8%), whilst 11 (64.7%) dogs underwent implant removal. Samples from removed implants for bacteriological culture were collected from 6/11 (54.5%), none of which yielded bacterial growth.

### 3.4. Bacterial culture results and antimicrobial susceptibility testing

A total of 91 bacteriological culture results collected from 80 patients were available for analysis. In 69 (86.3%) dogs, a single culture was performed, and paired cultures in 11 (13.8%) dogs. Overall, in 69.2% (*n* = 63/91) of the samples bacterial growth was detected. Sixteen different bacterial species (Table 5) were isolated with *S. pseudintermedius* being by far most frequent (*n* = 37) followed by *S*. *aureus* (*n* = 10).

**Table 5:** Bacterial species and number of multidrug resistant bacteria isolated from 91 bacteriological culture collected from 80 dogs treated with TPLO.

**Bacterial species detected**	**Total number of isolates**	**Number of multidrug resistant isolates (%)**
*S*.*pseudintermedius*^a^^,^ ^d^^−^^*f*^	37	13
*Staphylococcusaureus* ^b^	10	5
*Pseudomonasaeruginosa* ^a^	4	0
*A*.*baumanii*^b, d^	2	2
*E*.*coli*^f, g^	2	1
*Streptococcusdysgalactiae* ^e, g^	2	0
*Mixedbacterialflora* ^c^	2	Not applicable^*^
*K. pneumoniae*	1	1
*Clostridiumperfringens* ^b^	1	0
*Streptococcuscanis* ^f^	1	0
*Enterococcus faecalis*	1	0
*Pasteurella canis*	1	Not applicable^*^
*Pasteurella multocida* subsp. multocida/septica^d^	1	Not applicable^*^
*Proteusmirabilis* ^c^	1	0
*Pseudomonas alcaligenes*	1	0
*Corynebacterium auriscanis*	1	Not applicable^*^

Overall positivity rate was 69.2% resulting in 63 positive bacteriological cultures.

a, b, c, d, e, f, g Multiple bacteria isolated from one culture. Isolates marked with the respective superscript were isolated from the same sample.

* Antimicrobial resistance testing not performed.

In cases with a single bacterial culture, samples were submitted either when surgical site infection was first suspected (wound swabs/aspirates/tissue samples) or during removal of TPLO implants (screw, wound swab, tissue samples). Bacterial growth was detected in 72% (*n* = 50/69) of the samples. In 11 dogs, bacterial culture was performed when SSI was first suspected as well as during implant removal, which allowed pairing and comparison of results (Table 6). Paired samples were positive in six dogs. In all six cases, bacterial species isolated as well as the pattern of antimicrobial resistance were identical for both samples.

**Table 6:** Bacterial species and antimicrobial resistance pattern, antimicrobial treatment, and occurrence of complicated infection in 11 dogs with paired bacterial culture.

**Dog No**.	**Bacterial species isolated from swap/aspirate/ tissue sample**	**Bacterial species isolated from implant**	**Antimicrobial Resistance Pattern according to AST**	**Antimicrobial treatment following AST**
1	*S. pseudintermedius*	*S. pseudintermedius*	CLI-ERY-PEN-TET	ENR-CE
2	*S. pseudintermedius*	*S. pseudintermedius*	PEN	CLI
3	*S. pseudintermedius (MDR)*	*S. pseudintermedius (MDR)*	CHL-ERY-KAN-TET-GEN-SXT-OXA-ENR-MRB-AMS-PEN-CLI	None
4	*S. pseudintermedius* (MDR)	*S. pseudintermedius* (MDR)	ERY-KAN-GEN-SXT-OXA-ENR-MRB-AMS-PEN-CLI	None
5	*S. pseudintermedius* (MDR)	*S. pseudintermedius* (MDR)	ERY-KAN-GEN-TET-SXT-OXA-MRB-AMS-PEN-CLI	CHL
6	*S. aureus (*MDR)	*S. aureus* (MDR)	PEN-OXA	CLI
7	*S. aureus* (MDR)	sterile	PEN-OXA	CLI
8	*E. coli* (MDR)	sterile	AM-AMC-ENR-GEN-PIP-MRB-CHL-SXT-TET	CE
	*S. dysgalactiae*	sterile	LEV	
9	*S. pseudintermedius* (MDR)	sterile	CLI-ERY-PEN-TET-GEN-OXA	MRB
10	Sterile	sterile	Not applicable	Not applicable
11	Sterile	sterile	Not applicable	Not applicable

AST, antibiotic susceptibility testing; S, *Staphylococcus*; MDR, multidrug-resistant; CE, Cefalexin; CLI, clindamycin; ERY, erythromycin; TET, tetra-cycline; PEN, penicillin; GEN, gentamicin; ENR, enrofloxacin; CHL, chloramphenicol; KAN, kanamycin; SXT, trimethoprim + sulfamethoxazole; OXA, oxacillin; MRB, marbofloxacin; AMS, ampicillin + sulbactam; AM, ampicillin; AMC, amoxicillin + clavulanic acid; PIP, piperacillin; LEV, levofloxacin.

A total of 22 MDR bacteria were isolated after 21/769 TPLO (2.7% overall, 30.8% SSI). Thirteen of 37 (35.1%) *S*. *pseudintermedius* and five of ten (50.0%) *S*. *aureus* were classified as MDR. Other MDR isolates included *Acinetobacter baumanii* (*n* = 2), *Klebsiella pneumoniae* (*n* = 1) and *Escherichia coli* (*n* = 1) (Table 5). After one TPLO, two different MDR bacteria (*S*. *aureus* and *A. baumanii*) were isolated from the same surgical site.

## 4. Discussion

The purpose of this study was to investigate SSI after TPLO, associated risk factors, isolated bacteria and respective antimicrobial resistance pattern, as well as the course of treatment. Postoperative SSI rate was found to be 8.5%, which is in line with previously published data (4, 8, 9, 29, 30). Similarly, the incidence of 2.7% MDR infections is comparable to the literature (8, 9, 11). We could confirm previous findings that German Shepherd Dogs have a higher risk to develop SSI after TPLO (13, 17, 30).

The incidence of major SSI of 80.0% in the subpopulation of patients diagnosed with a SSI is higher than previously reported (4, 5, 9, 31). The higher incidence of major SSI could be partially explained by different definitions for SSI and what is considered a major SSI. Since our implant removal rate (64.6%) were similar to other studies, the higher rate of major SSI could be attributed to cases that underwent a surgical intervention as part of the treatment of active infection. Therefore, this finding could indicate a more aggressive approach to treatment of SSI, prompting more frequent surgical intervention such as reopening and debridement of the wound and the resulting in the classification of a major SSI.

The rate of SSI subjectively shows a trend toward a higher incidence in the latter study period (Table 2). Similarly, MDR bacteria were more frequently isolated. This finding is conclusive, since we found a strong correlation between number of SSI and isolation of MDR bacteria per year. In both human and veterinary medicine, bacteria resistant to many available antibiotic compounds are considered one of the largest threats. Ironically, the use of antimicrobials itself is one of the main drivers behind this emerging problem, since exposure to antibiotics leads to selection and spread of resistant strains (18, 32). For TPLO surgery, perioperative antibiotic prophylaxis is considered a standard protocol but given the emergence of MDR bacteria, could be reconsidered in an effort to combat future infections. Prospective, long-term studies would be necessary to investigate the benefits and risks of this approach.

Our finding, that *Staphylococcus* spp. was dominant among isolates from SSI following TPLO, is in accordance with previously reported study results (4, 10, 12, 20). Analysis of AST of *Staphylococcus* spp. revealed high detection rates of MDR *S*. *pseudintermedius* and *S*. *aureus* (37.1% and 50.0%). In comparison, the prevalence of methicillin-resistance amongst isolated *Staphylococci* from infection sites after TPLO reported in literature varies between 20% and 40% (8, 10, 11, 16, 17, 20, 21). Other than beta-lactam antibiotics, clindamycin was frequently utilized for empiric therapy of suspected SSI. Resistance rates against these classes of antimicrobials amongst *Staphylococcus* spp. isolates in our population were shown to be rather high. For multiple reasons, these results should be interpreted with caution and proposing a general recommendation regarding the choice of perioperative antimicrobial prophylaxis and empiric antibiotic treatment should be avoided. Firstly, use of perioperative antimicrobials could have influenced these findings by selection of resistant isolates. Similarly, some bacterial culture samples and respective AST were obtained during implant removal, at which point previous antimicrobial treatment might have influenced culture results. Moreover, comparison of our results with previous studies suggests, that dominant bacterial species as well as patterns of antimicrobial resistance may vary greatly (4, 10).

When comparing bacterial isolates before and after implant removal in dogs with paired bacterial cultures, it was found that isolates, as well as antimicrobial resistance patterns, were identical within each dog, which contradicts a previous report (10). Furthermore, although antimicrobial therapy was appropriately adjusted with regards to AST, results after first positive cultivation in four cases demonstrated, that bacteria withstood treatment. From all four aforementioned cases, *S. pseudintermedius* (*n* = 3) or *S. aureus* (*n* = 1) spp. was isolated; a genus of bacteria known to form biofilm that impedes host response and curative effect of antimicrobials (24).

Our finding, that the use of non-locking vs. locking implants was protective against surgical site infection is in contrast with previous reports. Experimental and clinical studies have suggested a protective effect of implants using locking technology (14, 33–35). Locking implants are believed to lower infection rates by protecting the periosteal blood supply, provide more stable initial fixation and limit soft tissue dissection required for adequate implant placement (34, 36). The somewhat conflicting result of this study might be explained by the distribution of usage of non-locking implant and varying incidence of SSI during the study period. While 71% (*n* = 101/124) non-locking implants were used in 2005 and 2006, postoperative infection rates were found to only 3.5% vs. 9.6% during the later study period (Table 2). Considering that scientific evidence points in the opposite direction, it is unlikely that more frequent usage of locking implants caused the surge of SSI incidence. Instead, we hypothesize that other factors led to an increase. For example, the incidence of MDR bacteria in the later study period was higher, which might have overcome the beneficial effect of locking implants. Nevertheless, to our knowledge, a protective effect of locking implants for TPLO in a clinical setup has only been suggested for dogs weighting >50 kg (14, 35). Therefore, additional research in this field is needed to clarify this question.

We found that patients that had previous TPLO on the contralateral stifle had higher odds of postoperative infection. To our knowledge, this has not been reported before. In human medicine, patients that underwent previous surgery have been shown to be at greater risk for surgical site infection with methicillin-resistant *S. aureus* (37, 38). Similarly, in veterinary medicine, carriage of methicillin-resistant *S. pseudintermedius* has been associated with previous hospital stay and exposure to antimicrobials (39, 40). Nazarali et al. (11) have suggested, that carriage of methicillin-resistant *S. pseudintermedius* is a risk factor for development of SSI after TPLO. Nevertheless, since the number of dogs that meet these specific criteria in our study population is relatively low, investigation of associations between previous surgery/hospital stay and isolation of MDR-bacteria from an SSI site was not possible. Furthermore, we only included previous TPLO, but not previous surgery in general as a possible risk factor.

In total, 2.2% (*n* = 17) patients evaluated for ongoing lameness after TPLO were classified as having persistent lameness of unknown origin. Even though bacterial samples from implants of 6/11 (54.4%) were collected and did not yield bacterial growth, it is possible that the infection rate could have been underestimated due to a misclassification bias. On the other hand, lameness could have been caused be factors not directly related to TPLO surgery, such as progression of degenerative joint disease of the stifle. This applies mainly to cases, where the diagnosis was made several months after the surgery.

There are several limitations of this study. In respect to its retrospective nature, we wholly depended on completeness and accuracy of data collected from electronic medical records. Furthermore, subsequent patient information was not always available, due to owners electing to perform rechecks in a different practice. Additionally, the definition of SSI used was very narrow and would not include cases of early-stage SSI which only show signs of inflammation. For this reason, complication rates may have been underestimated.

We can conclude that incidence of major SSI was found to be high in the study population but was not associated to isolation of MDR bacteria. In contrast, isolation of MDR bacteria was correlated with SSI rates in general. Additional research is needed to investigate a possible link between previous hospital stay and use of non-locking vs. locking implants on risk for SSI after TPLO in general and SSI involving MDR bacteria in particular.

## Data availability statement

The raw data supporting the conclusions of this article will be made available by the authors, without undue reservation.

## Author contributions

BH, UR, GO, and FF designed the study. BH and GO collected the data. GO provided data on bacteriological examination of the SSI cases discussed. BH was responsible for the statistical analysis of the collected data. UR supervised data collection and analysis. BH, UR, and GO drafted the manuscript. All authors critically revised the manuscript and approved the final version.
